# Making Connections that Count – a Case Study of the Family Referral Service in Schools Program on the Central Coast, New South Wales, Australia

**DOI:** 10.5334/ijic.6998

**Published:** 2023-02-09

**Authors:** Hazel Dalton, Jamin Day, Tonelle Handley, Angela Booth, Alan Hayes, David Perkins

**Affiliations:** 1 Rural Health Research Institute, Charles Sturt University, Orange, NSW, Australia; 2 School of Medicine and Public Health, College of Health, Medicine and Wellbeing, University of Newcastle, Callaghan, NSW, Australia; 3 Healthy Minds Research Program, Hunter Medical Research Institute, Newcastle, NSW, Australia; 4 School of Humanities, Creative Industries, and Social Sciences, College of Human and Social Futures, University of Newcastle, Callaghan, NSW, Australia; 5 University Library, University of Newcastle, Callaghan, NSW, Australia; 6 Macquarie University, Macquarie Park, NSW, Australia; 7 Mental Health Policy Unit, Health Services Research Institute, University of Canberra, Canberra, ACT, Australia

**Keywords:** vulnerable youth, formative evaluation, integrated care, Australia, case study

## Abstract

**Introduction:**

Adverse childhood experiences (ACEs) are associated with health and social problems in later life, with an early intervention highly desirable for better outcomes.

**Description:**

The Family-Referral-Services-In-Schools (FRSIS) is an early-intervention case management program for children and families with complex unmet needs, providing access to family support, housing, mental health care, and/or drug and alcohol services. The in-school trial setting was aimed at improving service uptake which was low in its community counterpart.

**Discussion:**

FRSIS was a well-regarded intervention that reduced barriers to access for vulnerable families. The school setting and non-government agency service provision led to increased acceptability and trust. The program reached 5% of the student population. Support was tailored to family need, which was often complex and involved both children and caregivers. Initially, the multi-agency partnership and governance oversight group championed the service and enabled the pilot to be established, however funding uncertainty and competing priorities saw leadership support ebb away despite operational success.

**Conclusion:**

The FRSIS model breaks down numerous barriers to accessing care for vulnerable families by its generalist nature and tailored approach and represents a high-trust approach to brokering appropriate care. Consistency in leadership support was a missed opportunity for program sustainability.

## Introduction

Intervening early to address health and social issues in childhood is widely considered to have lifelong benefits. Investment in children leads to healthier adults, better able to reach their potential to positively contribute to their community [1,2,3,4,5]. Adverse childhood experiences (ACEs) are associated with poorer health and social problems in later life, including increased risk of physical diseases, poor mental health outcomes, substance abuse and suicide [6,7]. ACEs are considered hallmarks of vulnerability and include adversities such as abuse, neglect, and household dysfunction.

ACEs may affect subsequent generations producing cycles of disadvantage. Poor educational outcomes result in few employment opportunities, reduced income and potential poverty [8]. Growing up in neighbourhoods where poverty, unemployment, housing instability and domestic violence are high exposes children to an increased risk of ACEs [9], thus behaviours become entrenched and passed on. Effective early intervention has the potential to minimise the negative effects of ACEs on long-term health and wellbeing [10,11].

In New South Wales (NSW), the policy context to support vulnerable children and young people was influenced by a 2008 inquiry into children in protective services [12]. In response, the NSW Government implemented several initiatives aimed at improving children and young people’s wellbeing [13]. These focused on effective delivery of health and social services, with a whole-of-government integrated approach and a focus on early intervention. This included the establishment of an early intervention case management program – the Family Referral Services (FRS) program – for children and families, where child protection services are not required, but needs are complex and unmet. The FRS program supports families through family support, housing, mental health care, and/or drug and alcohol services. An evaluation of FRS noted that whilst an effective service, uptake was relatively poor [14]. Furthermore, in a subsequent review of children in out-of-home care, the key drivers of children into care were mental health issues affecting parenting capacity, domestic and family violence that make home life unsafe and parental drug and alcohol abuse [15,16]. Addressing these issues and ensuring access, engagement and educational attainment were the imperative of the next Government response. In parallel with the reforms charted above, the NSW Department of Health advocated a whole-of-government approach to the integration of health care [17], with investments in a series of demonstrator programs including on the Central Coast [18].

These health and social care reform activities, coupled with the premise that engagement in education has broader implications for the wellbeing of children and young people and into later life, led to a version of the FRS program being trialled in NSW schools – Murrumbidgee [5] and independently on the Central Coast, the subject of this evaluation, initially piloted as part of the Central Coast Integrated Care Program [18]. This evaluation was co-commissioned by the same multi-agency group that oversaw the operational governance of the FRSIS program.

In this case study, we describe the Central Coast Family Referral Service In Schools (FRSIS) program with details of the intervention, its implementation and evidence of the program’s impact. The key lessons derived from the study are discussed.

## Ethical approval and case study approach

In 2018, the author group (research team) were commissioned to conduct a formative assessment and impact evaluation of the FRSIS program by the multi-agency oversight group. By this time FRSIS had been in pilot in some schools since 2016. Ethical approval was granted in 2019 by: Hunter New England Human Research Ethics Committee (2019/ETH08686); State Education Research Applications Process (SERAP2019187); Central Coast Local Health District (Site-specific 0519-016C) and University of Newcastle (recognition H-2019-0168).

Study design: A collaborative approach between the research team and the FRSIS multi-agency oversight group and stakeholder network was used to design the evaluation. This was done to represent the diverse stakeholder objectives in supporting the program, to build consensus on the evaluation approach and to identify and appraise existing supporting data sources. The stakeholders represented the organisations involved in the planning and implementation of the program (i.e., schools, commissioned Non-Government Organisation (NGO), local Primary Health Network, local departments of health, social care and education, and other health and community service providers). A series of three workshops were conducted by the research team with key stakeholders, one face to face from each learning community (n = 9 and n = 12 for communities A, and B and C respectively) and a virtual workshop regarding data linkage feasibility (n = 7).

The evaluation questions to explore the program’s implementation and its outcomes are outlined in **Box 1**.

**Box 1 Primary evaluation question and sub-questions**

The primary evaluation question was: ***How is the Family Referral Service in Schools program operating to contribute to the objectives of the multi-agency oversight group?***

This was explored through the following sub-questions:

1. What has been the history of implementation of FRSIS?
2. Who uses FRSIS?
3. Is there evidence of changes likely to be related to improved educational outcomes for students due to the implementation of FRSIS?
4. Is there evidence of FRSIS connecting students and their families to services? What are the benefits of doing so?
5. Is there any evidence of changes in school staff experiences due to the implementation of FRSIS?
6. Is there evidence that FRSIS has produced other, secondary gains?
7. Is there any evidence of negative consequences resulting from the implementation of FRSIS?

A pragmatic mixed-methods approach was adopted to make best use of operational data, the views and perspectives of key stakeholders and a review of program documentation. The research team also attended seven FRSIS multi-agency oversight working group meetings over the project period (2018–2019) and sought verbal and written feedback on various stages of the evaluation including protocol design.

It was determined that interviews with students and their families who used the service would not be conducted due to concerns raised by both FRSIS staff and teachers.

Notably, a key ambition to link FRSIS data to other government data sources to yield in-depth insights was the subject of a workshop. There were recording procedure limitations for FRSIS data and in-depth consultation with the NSW Government Data Analytics Centre, which has responsibility and capacity for such interdepartmental linkage, revealed it would be cost-prohibitive for this project and unlikely to yield an ongoing capacity to follow up on FRSIS referral outcomes without substantial data infrastructure investments. Thus, the key indicators of program performance reported are program activity, and teacher and stakeholder reports.

Qualitative data were collected by reviewing 21 program-related documents, five semi-structured telephone interviews (10% response rate) and six example case histories constructed by the commissioned NGO to provide anonymised accounts of program users’ experiences and outcomes. The purposive sample for interview and survey included school staff (n = 32) and those from affiliated organisations (n = 20), across leadership and operations roles. The interviews questioned participants’ understanding of the program and its operation. Interviews were audio-recorded and transcribed with participant permission. The low response rate and the lack of in-depth quality data within the provided documents made systematic thematic coding analysis impractical. Instead, the different qualitative sources were examined for broad insights. Quantitative data collection included a de-identified FRSIS operational dataset (2016–2019, n = 429 referral records) and 14 surveys (27% response rate) to assess both implementation progress and perceived outcomes of the program. The survey participants were asked to indicate their level of agreement, using a 5-point Likert scale, with statements about the program’s implementation, efficiency, effectiveness, and benefits (a ‘don’t know or not applicable’ response was provided). The anonymous survey asked only for participants to indicate their current roles. Analyses of these data were limited to descriptive statistics. Microsoft Excel and R [19] were used to collate, describe, and visualise data.

In the case study, data from different sources are presented and discussed together. Where the source is not stated, information is from stakeholder documents or records supplied. The school-based family support workers from the commissioned NGO were referred to by various titles, ‘Family Engagement Worker’ is used here, and ‘parent’ is used to refer to anyone in a guardianship role.

## Description of the care practice

### Development

The Family Referral Service in Schools (FRSIS) program was designed to connect families, with a school-aged child (5 to 18 years) at risk of disengaging from education, with the health and social services they need. Between 2016 and 2018, the program was introduced into schools in three learning communities in the NSW Central Coast region. The program was part of a suite of integrated care initiatives implemented under the Central Coast Integrated Care Program (CCICP) [18].

The program was part of the response to the area’s high proportion of vulnerable children and young people and a state-wide intention to improve family health and social services [20,16]. In terms of estimated developmental vulnerability, the Australian Early Development Census (AEDC) assesses developmental risk across a number of domains, physical, social, emotional, language and communication. In 2018, 9.8% of children on the Central Coast were estimated to be vulnerable on two or more AEDC domains [21]. The Central Coast has several issues associated with cycles of disadvantage, including high rates of substance abuse, domestic violence, youth unemployment, teenage pregnancy, and low school completion rates [22], in a context where services were both siloed and overloaded by demand [16]. Hence, a more integrated and coordinated approach was thought necessary to facilitate service navigation [20,16].

The Central Coast FRSIS program was an evidence-informed (literature review and integrated care expert engagement) adaptation of the FRS program into the school context to better facilitate access to the health and social care needs of vulnerable children and their families. A detailed program logic was developed for the pilot in consultation with the multi-agency oversight group.

### Implementation

The program began in Learning Community A in 2016, with around 4,250 students (two high schools, five primary schools). In 2018, Learning communities B (two high schools, five primary schools) and C (one high school, three primary schools), with around 8,460 students in total joined. Some schools opted out of participation. Nearly all participating schools served socially disadvantaged communities as measured by the Family Occupation and Employment Index (FOEI). FOEI is used by the NSW Government for needs-based resource allocation. The commissioned NGO implemented the program with school support. Table 1 details the participating schools, including the FOEI score.

**Table 1:** Profile of participating Learning Communities (as of May 2018)*.


LEARNING COMMUNITY	SCHOOL	NO. OF STUDENTS	PARTICIPATION IN FRSIS

**Learning Community A** *Approximately 4,243 students*. FOEI** Range of 80–127	School 1 (Years 7–9, FOEI 127)	878	Yes, October 2016

	School 2 (Years 10–12, FOEI 119)	650	Yes, August 2017

	School 3 (Years K–6, FOEI 120)	537	Yes, November 2017

	School 4 (Years K–6, FOEI 124)	487	Yes, November 2017

	School 5 (Years K–6, FOEI 103)	466	Yes, December 2017

	School 6 (Years K–6, FOEI 93)	768	Yes, February 2018

	School 7 (Years K–6, FOEI 80)	457	No

**Learning Community B** *Approximately 4,459 students*. FOEI Range of 126–150	School 8 (Years 7–12, FOEI 137)	832	No

	School 9 (Years 7–12, FOEI 126)	963	Yes, March 2018

	School 10 (Years K–6, FOEI 135)	574	Yes, December 2017

	School 11 (Years K–6, FOEI 140)	551	Yes, February 2018

	School 12 (Years K–6, FOEI 150)	609	Yes, February 2018

	School 13 (Years K–6, FOEI 143)	382	Yes, March 2018

	School 14 (Years K–6, FOEI 131)	548	Yes, March 2018

**Learning Community C** *Approximately 1,764 students*. FOEI Range of 116–135	School 15 (Years 7–9, FOEI 123)	717	No

	School 16 (Years K–6, FOEI 135)	237	Yes, March 2018

	School 17 (Years K–6, FOEI 118)	375	Yes, March 2018

	School 18 (Years K–6, FOEI 116)	435	Yes, May 2018


* Data sources: FRSIS documentation and My School website https://www.myschool.edu.au/, coded here for anonymity. ** Family Occupation and Education Index (FOEI) Scores greater than 100 (the mean) indicate educational disadvantage related to socio-economic background.

FRSIS was managed by a team-leader employed by the commissioned NGO. Up to two Family Engagement Workers were employed on the Central Coast: one for Learning Community A (0.8 full-time-equivalent) and the other for learning communities B and C (full-time). Schools nominated staff responsible for referring students in need of help. Students were eligible if there was evidence of educational neglect, risk of disengaging from school or family welfare concerns (e.g. family violence, mental health, homelessness). However, engagement with other case management or child protection services were exclusionary criteria. With parental permission, the Family Engagement Worker assessed the whole family’s needs, the appropriate health and social services and any barriers to access. Support and strategies to link the family to services were then provided.

Key staff in schools and the commissioned NGO changed throughout implementation, but frontline program operation continued. Interview data revealed that the program was supported by clear processes covering personnel changes and absences that ensured smooth transitions. Furthermore, most survey participants agreed (9/14) that systems were in place that ensured efficient transitions between Family Engagement Workers.

**Figure 1:**
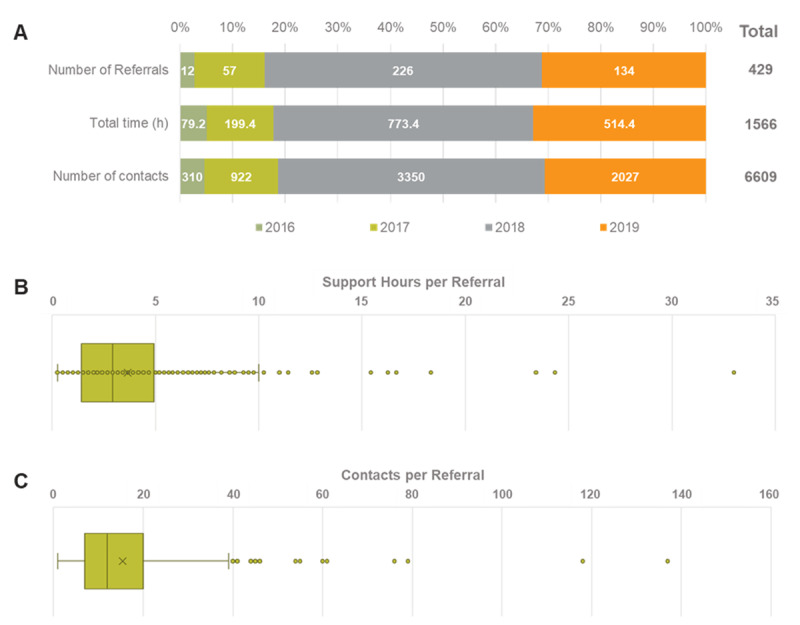
Profile of FRSIS referrals by year **(A)**, support hours **(A, B)** and contacts made **(A, C)**.

**Figure 2:**
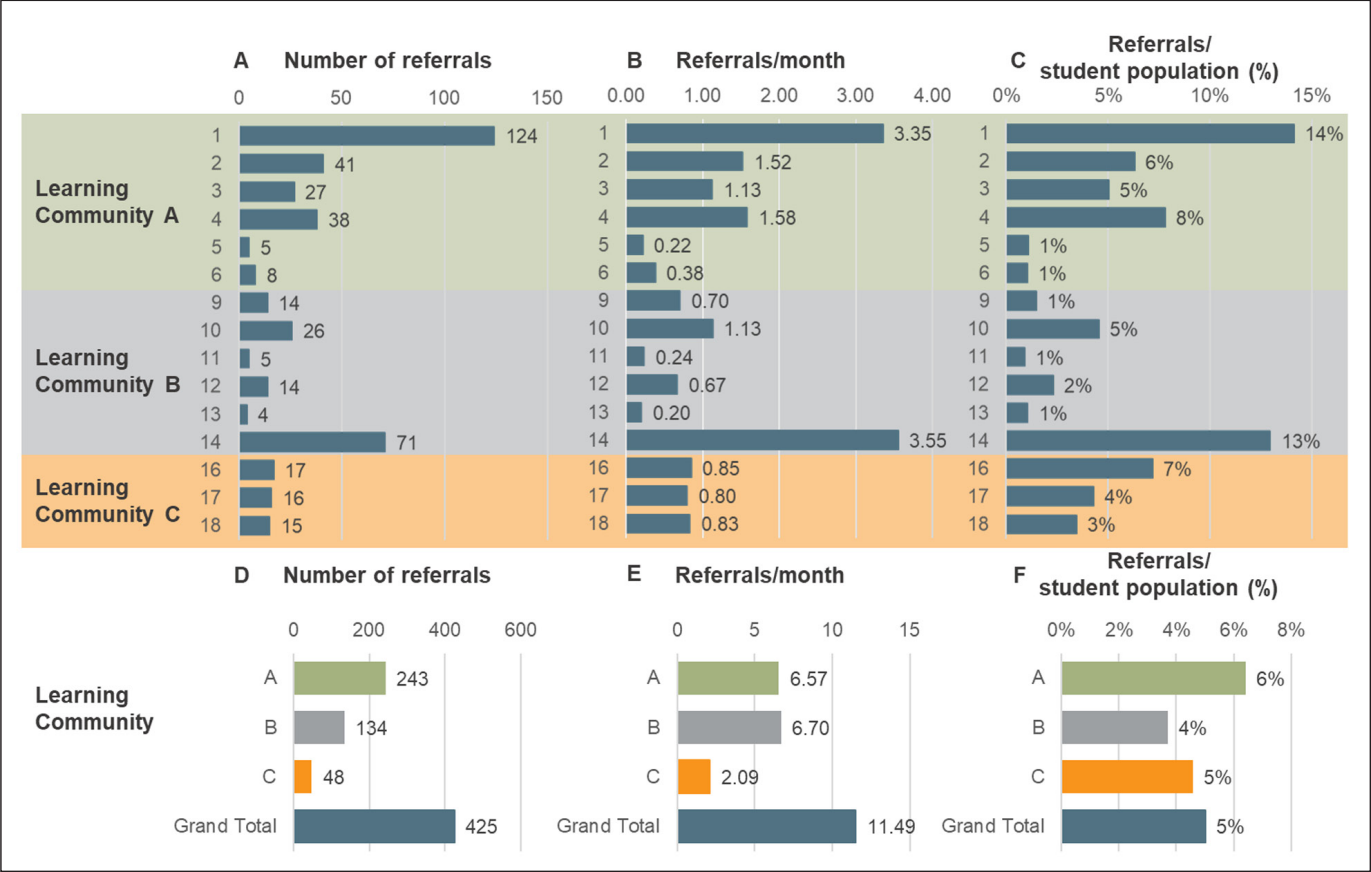
FRSIS referrals: total number* **(A, D)**, per month **(B, E)** and per student population (%, **C, F**) by school and Learning Community respectively. * We note that 4 referrals were made by Department of Education staff outside of schools, these have been omitted here for clarity.

Between August 2016 and November 2019, 392 families were referred to the service on 429 occasions (some families were referred more than once, Figure 1). This represents a reach of 5% of the total student population (ranging from 1% to 14% per school, Figure 2). A summary by year of the number of referrals made, with supporting engagement contacts and time spent supporting referrals are shown in Figure 1. The number of contacts and support time increased significantly in 2018 when Learning Communities B and C joined but decreased in 2019.

Program use also varied across schools (Figure 2). Strong program use was seen for two schools (Schools 1 and 14 at 14% and 13% respectively), whilst five schools had very limited use at 1% suggestive of varied implementation and maturation of FRSIS. This variation is use was not associated with the time of first referral or FOEI score. The schools with the highest number of referrals implemented FRSIS at different times (School 1 in September 2016, School 14 in March 2018 and School 5 in November 2017). Moreover, the schools serving the most disadvantaged communities (Schools 12, 13 and 11) and those with the least disadvantaged (Schools 5 and 6) referred the same proportion of their student population (1%). The level of engagement with the program appeared to be more important. Locally, the program was first piloted at School 1 and the Principal undertook a lead role in implementation in other schools as did the Principal of School 14.

**Figure 3:**
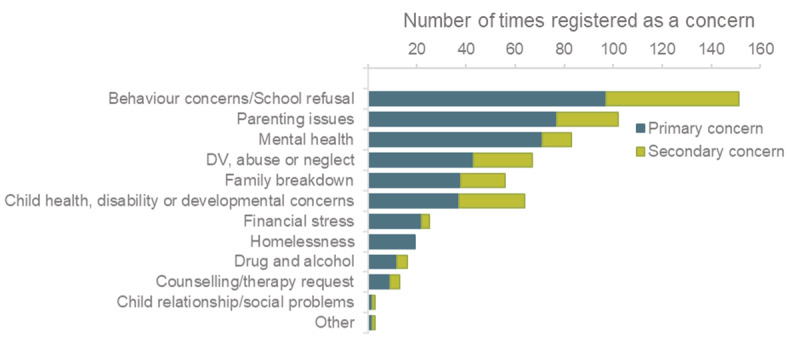
Primary and secondary reasons for referral to FRSIS.

A primary reason for referral was recorded for each case. The most common primary reason was behavioural problems including refusal to attend school. Parenting and carer issues were the second-most common, followed by mental health concerns; domestic violence, abuse or neglect; and family breakdown. In about 40% of cases, a secondary concern was also registered; and additional concerns were recorded in less than 10% of cases. Behavioural problems, child health, disability or developmental concerns, parenting issues and domestic violence, abuse or neglect were the most common secondary concerns (Figure 3). Interviews and case histories also indicated that behavioural concerns were the most common reason for referral, particularly educational disengagement or poor functioning at school as concerns.

Interviewees reported that referred families were primarily experiencing issues requiring complex social care (e.g. financial problems, family violence, family relationship problems, substance misuse and parenting problems). This was affirmed by the documentation and case studies. It was not uncommon for grandparents to have stepped into the parenting role. These grandparents often had complex health care needs of their own and were without support and respite care. However, interview participants reported a great deal of diversity amongst the presenting issues of students and their characteristics, although common characteristics of families were that they didn’t know how to, or were unable to, access services themselves.

After the family assessment, the Family Engagement Worker made outbound referrals to appropriate services. Support was provided by initially contacting the services, educating parents and students about what was available and/or taking them to services. Depending on need, the coordination of outbound referrals was through face-to-face meetings (n = 43), telephone (n = 112), home visits (n = 152), and/or provision of information (n = 130). Non-referral information (e.g. education and support resources) was provided to families where appropriate (n = 119). When families engaged with services, most cases were closed, with the ongoing care being undertaken by the receiving services. Case histories showed that sometimes the Family Engagement Worker had multiple unsuccessful attempts to engage the family, with one example taking over six weeks.

Program records showed most families were being referred to services and/or provided with information. Of the 429 occasions of service, 58.8% received an outbound referral. Service types that families were referred to are summarised in Table 2. Most referrals (50%) were made to social care types of services, with the remainder to health (26%), education (2%) and other (20%) services. Families were recorded to have accessed the service for 65.7% of outbound referrals. However, some families may have accessed their outbound referral without notifying the Family Engagement Worker. The primary support for those not receiving an outbound referral was the provision of information.

**Table 2:** Types of services for outbound referrals.


TYPE OF REFERRAL SERVICE		*n* 417

**Social Care –** including departmental initiatives, other counselling, drug and alcohol services		211

Parent/family support	92	

Other counselling	42	

Housing	22	

Welfare	37	

Centrelink	9	

Gambling counselling	1	

Child Protection Helpline	5	

Drug and alcohol	3	

**Health**		110

Mental health counselling	67	

GP	11	

Health	25	

Paediatrician	6	

Speech Pathologist	1	

**Education**		10

Education	9	

Vocational Training Provider	1	

**Other –** including legal, sport and recreation and disability services		86

Sport and recreation	16	

Legal support	11	

Disability	29	

Other (not specified)	30	


In interviews, the only circumstance under which FRSIS was said to not work was if the family refused the engagement. However, from operational data, no outbound referral was received on 41.2% of occasions of service and of those that did receive a referral, 34.3% were not known to have accessed the service. The main reasons for not providing a referral are outlined in Table 3. In most cases, it was confirmed that the family’s needs were being addressed (already linked with a service [exclusionary criterion as outlined above], information was provided or the family self-referred). For 49 occasions of service (11.4%), the family declined help, could not be contacted, or did not meet the program’s criteria and hence were unable to be helped. For the remaining occasions of service (about 4%), it would appear there were broad system failures (further assessment required, client deteriorated and couldn’t access the referral or there was no appropriate service).

**Table 3:** Summary of main reasons provided for not referring families to other services.


REASON	N

Client did not meet the service criteria – Family already linked with service (including having an open plan with CSC*)	71

Information only given	34

Family declined	25

Family not able to be contacted	20

Family will self-refer	12

Further assessment required	12

Client deteriorated and couldn’t access referral	2

No appropriate service	1


*CSC = Community Service Centre for Department of Community and Justice.

Whilst operational data provides a snapshot of care provision, case histories provide an opportunity to see the flexible nature of support and the difference the referral service made to families. **Box 2** shows two illustrative case histories that were based on the six de-identified case histories provided. These show the complexity of family assessments to identify the need and the support required, leading to multiple referrals to health and social care to support the whole family.

Box 2 Illustrative case histories*

Case History 1

The school noticed Jane’s attendance had been low for the past two years. When at school, she frequently displayed aggressive outbursts and defiant behaviour. The family assessment revealed that Jane’s father John had severe depression and her two siblings had special care needs.

The Family Engagement Worker arranged a referral for all the children to a paediatrician with a short waiting list, negotiated reduced fees and accessed the NGO’s brokerage funds to financially assist the family with medication not available on the Pharmaceutical Benefits Scheme (government subsidy).

Jane was diagnosed with anxiety and a mild intellectual disorder. Referrals were made to the NGO’s disability team to further assess and discuss the support needs of the family, including the mental health needs, and to assist with a National Disability Insurance Scheme application. Peer support via an NGO-led community mental health program was arranged for John.

Since the referral, Jane’s school attendance improved as did her behaviour at school and at home. The school attendance of her siblings was also more consistent. The family accepted all support offered and continues to work with longer-term support services. Her mother Anne reports feeling confident that they are getting the right support and that they are setting a good example for their children.

Case History 2

The school was concerned about Jack’s sad and withdrawn emotional state. He was increasingly unable to control his emotions, having violent outbursts. A family assessment revealed that Jack’s parents had recently separated and his older brother, Michael, also had very low attendance. Michael had been refusing to get out of bed and had run away from home in the past. Their mother Judy was struggling to manage. She was casually employed and the shared housing arrangement that she and her children were in was inadequate. There were also issues around domestic violence.

Judy was reluctant to engage with health services. Moreover, Tom, the father, was highly resistant and hostile. The family’s need for ongoing support made them ineligible for a short-term general family support program. However, their support needs also did not meet the ‘Risk Of Significant Harm’ (ROSH) criteria which would have enabled support from the social services department.

Nevertheless, the Family Engagement Worker collaborated with a homeless case management service to arrange more suitable housing, and Judy and her children were successfully relocated. They were able to access support from extended family and were connected with the FRS in their new location.

*Amalgam of de-identified case histories provided; names are fictional.

### Evaluation

Operationally, the program was used by students from all participating schools, but with varied uptake. All interviewees regarded the program extremely positively, emphasising that this low-cost initiative provided a non-threatening approach to assist families to access much-needed services. This was echoed in the survey, with all 14 respondents agreeing or completely agreeing that, overall, the program was working well.

#### Benefits for students and families

All interviewees said the program successfully supported students and their families to access services. They reported that FRSIS enabled identification of students in need within schools, and whole family support as a consequence. This was thought important since the main support issues often lay with the parents. Most survey respondents also believed the program helped families to access services (10/14) and more families accessed services because of FRSIS engagement (10/14).

In workshops, it was reflected that the Family Engagement Worker could avoid the need for families to continually retell their stories and problems by liaising with the different service providers. Interviewees said that the bridge to services provided ensured that families quickly felt that something was being done, even if there was a waiting list. Interview and case study data showed other barriers to care access were lowered through sourcing financial support for service fees and transport to appointments when necessary.

Due to confidentiality concerns and the burden that de-identified data provision would place on resource-stretched schools, student attendance and behaviour records were not obtained. Hence, interview and survey data were relied on to assess whether program participation led to improved educational and behavioural outcomes. Interviewees were uncertain about whether the program had improved the educational outcomes of students. However, it was thought that school attendance had improved. Survey responses indicated a perception (10/14) that the program had improved educational engagement and attainment of students. Case histories demonstrated improvements in school attendance after appropriate support was received. Better behaviour at school and at home was also apparent. Similarly, interviewees perceived that the behaviour of students, who had behavioural problem-related referrals, had improved. Around half (8/14) of survey participants agreed that the program had resulted in improvements to students behavioural and emotional well-being, whilst two disagreed.

#### Benefits for schools and staff

Interviewees perceived that FRSIS relieved some of the burden on the school staff in addressing behavioural issues and supporting student well-being. School counsellors and headteachers were reassured that student welfare was being addressed. Furthermore, this work was done more effectively and efficiently because of the Family Engagement Workers’ specialist knowledge of appropriate and available services. School staff interviewees reflected greater job satisfaction. However, just under half of school staff survey respondents (4/10) agreed that FRSIS had improved their overall work and/or teaching experience; two disagreed.

School interviewees believed FRSIS had fostered better relationships between themselves and parents by providing a non-threatening opportunity for having a conversation with parents in need of help. These participants believed that the program demonstrated a more holistic concern for the family’s welfare and not just a concern about educational performance. The view that the program had engendered an improved parental attitude toward the school was also expressed in the workshops. In surveys, just over half of school staff who completed the survey (6/10) agreed that their relationship with referred families had been improved by the program; two participants disagreed. Documents also suggested communication between schools and parents was improved.

In workshops, it was suggested that FRSIS played a role in de-escalation of crisis incidents, avoiding the need for more punitive responses. However, school survey responses were equivocal, with the same number agreeing (4/10) as disagreeing (4/10).

#### Facilitators and barriers

Interviewees emphasised that delivery by an NGO enhanced the program’s acceptability with parents. The commissioned NGO was understood to be perceived by parents as less authoritarian and punitive compared to schools and government departments (social services and education). It was thought parents felt more at ease if a Family Engagement Worker was present at meetings with perceived authority figures from the social services department and the school.

Interviewees reflected that the placement of the Family Engagement Worker within the schools increased the likelihood that families would be seen face-to-face (compared to the general FRS program) and facilitated case reopening when necessary. Meeting face-to-face was considered better for establishing rapport and building a partnership with parents.

In interviews, the only circumstance under which FRSIS was said to not work was if the family refused the engagement. The reasons for parental refusal of help included a perception that FRSIS would not provide them the help they needed. Also, the community-based FRS program was available to parents not wanting school involvement. For other families, it was said they may not have been able to accept help and that this was to do with the intrinsic family capacities rather than program failure. The document review indicated that intrinsic barriers included longstanding disadvantages, mental health issues, family violence, unemployment and drug and alcohol misuse.

Family refusal of FRSIS was not interpreted as a failure of the program in interviews. It was thought the Family Engagement Worker’s engagement improved family knowledge of local service availability. Furthermore, it was said that families who refused FRSIS help had sometimes later reported they had accessed the services or considered alternative educational options (e.g. home-schooling or distance education) to address the issues identified. Furthermore, another interviewee explained that refusal of help was an opportunity to consider how else the family could be supported.

The main extrinsic barriers to families accessing services noted in documents and interviews were long waiting times and ‘closed books’ (services not taking new referrals). It was understood that some services had so much demand that only families coming through the social service department could access them. Documents and interviews also indicated costs were a barrier to service access, especially psychological services. A lack of General Practitioners, especially those that did not charge the patient, was also considered to be an issue. Transport to services was also a barrier, but it was overcome in at least some cases by services travelling to the families or FRSIS arranging transport to appointments.

#### Governance and funding

The Central Coast FRSIS Working Group oversaw the implementation and governance of the program. Two learning community governance groups were developed to aid implementation. We received limited documentation related to governance, documentation related primarily to the Working Group. Since the FRSIS Manager was at the FRSIS Working Group meetings along with multi-agency representatives including the education department, when they did meet, a considerable amount of operational oversight was conducted at this level with strong communication from the frontline staff and schools. Attendance diminished over time as the program funding deadline approached and refunding concerns were not allayed.

The FRSIS Working Group had at least seven staff changes during the evaluation period, including two project champions from health and education who oversaw the initial implementation. The NSW Health Integrated Care Manager role was vacant for over a year and staff worked in multiple roles or were on secondment. In addition, the loss of momentum of the Learning Community A Governance Group appeared to be linked to the lead Principal leaving.

Funding was considered by most interview participants to be the most critical issue to ensure sustainability. Initial funding was provided by the health district’s integrated care demonstrator funding, with the hope of multi-agency co-commissioning or that schools would purchase the service using discretionary funding. There was uncertainty by interviewees about how the program was funded and whether the funding would be ongoing, this was also reflected in survey responses. A minority agreed the program was current funding was adequate (6/14) and that future funding would be forthcoming (4/14). It was noted in documents that because State and Commonwealth systems were not set up for joint funding that the multiagency co-commissioning of the program had been challenging. With so many partners involved this problem threatened the program’s sustainability.

## Discussion

Operationally FRSIS functioned well with systems ensuring smooth transitions through staff changes. The number of referrals to FRSIS varied across schools, which appeared to be related to the school’s degree of program engagement and implementation maturity. The FRSIS aimed to provide support for families with children at risk of disengaging from education and connecting them to services were realised. FRSIS assisted families of students with behavioural problems, reduced school engagement and those where the welfare of the student was a concern. Indeed, FRSIS was perceived to be an effective means of helping families with complex needs, unable to access services without help, and who otherwise might fall through the net. In terms of considering integrated care access [23], FRSIS improved care access for families across the dimensions of needs identification, care-seeking, care-reaching and care use.

Moreover, school staff experienced benefits, reporting that FRSIS fostered the belief that students’ welfare was being taken care of effectively and efficiently and relieving them of that burden. It was also apparent that the program could improve school/parent relationships. It has been documented that parental involvement with their child’s education and school leads to better educational outcomes for the child [24,25].

The handling of student and parental problems by an outside non-government agency was also understood to improve program acceptability. However, it was valued because the service was within schools because it linked students in need to appropriate help and allowed face-to-face contact. It is difficult to conceive how families in greatest need of the kind of help that FRSIS provided would get to access the community-based version of the program.

The inability to easily access cross-agency linked data related to students and families involved in the program hampered a deeper assessment of FRSIS impacts and represents current systemic limitations. Thus it was not possible to measure longer-term potential gains (or early indicators thereof) in areas such as education and health outcomes. Thus, establishing data linkage systems that enable service-user outcomes to be tracked and planning to collect all relevant data pre-implementation is vital [26]. Even so, for those families helped, previous research suggests more positive educational, health and social outcomes are likely to eventuate [27,28].

FRSIS did not provide all families referred to it with an outbound referral, with some engaged with other services and others only required information. A minority, however, declined help or couldn’t be contacted. Declining service was not seen as a failure because it was an opportunity for increasing the family’s knowledge and some came back to FRSIS later.

Where FRSIS did not perform well was in the sustained engagement of the multi-agency Working Group. Meeting attendance declined over time, as the funding contract deadline drew near, and evaluation participation was very poor. The funding uncertainty and staff changes appear to have contributed to this. This decreased engagement likely impacted the ability to champion and secure ongoing program funding. Leadership is vital to the success of integrated care initiatives [29,30]. Recently, it was found that the governance and cross-agency partnership arrangements to deliver state-wide reform for children were found to be ineffective. The NSW Auditor-General noted that ‘*Their Futures Matter lacked mechanisms to secure cross-portfolio buyin and did not have authority to drive reprioritisation of government investment*’ [31]. The successes of this program appear to result from the effective early implementation in the school context and the commitment of frontline staff. We note that a reach of 5% of the total student population (ranging from 1% to 14% per school) would potentially cover almost half of the children at developmental risk across two or more AEDC domains on the Central Coast (9.6%). We note that not all students would require the pro-active support of FRSIS, and others would be engaged in more intensive case management support (FRSIS-ineligible). Short-term funding and premature funding withdrawal from successfully performing programs have been a recognised problem for Australian health and social services [32].

Other failures were outside the control of the program, including long waiting times and poor service availability, which thwarted families from receiving the needed services. These issues are common in the Australian health system and should be addressed [33,34].

The findings of this evaluation reinforce the prior findings [35] that FRS can operate successfully in Australian schools. Moreover, to our knowledge, FRSIS presented a unique multi-agency model with an NGO working within schools and in close cooperation to bring about outcomes that will mostly be measurable in the long term. We acknowledge there are many Australian and international programs with the same general aims to promote education engagement and school completion to bolster long-term wellbeing and break cycles of disadvantage [36,37,38,39,40,41,42,43]. However, these programs tend to be limited to specific issues and/or family demographics. FRSIS, on the other hand, is a broad-ranging flexible service for any family in need (that is not already being helped), regardless of their problems. The main lessons from this case learned are listed in **Box 3**.

## Lessons learned – implications for policy and practice

**Box 3 Lessons learned – implications for policy and practice**

- The FRSIS program worked well as a soft entry support for children and families who often fall through the support gaps due to service fragmentation, complex support journeys and long wait times. FRSIS Program data revealed a flexible and tailored approach to supporting families to provide a referral as well as a diverse range of services and information referred to across health and social care. Program data also revealed variance in uptake in schools suggesting uneven implementation success, to be expected of a program that had yet to reach maturity.
- Collaborative governance a strength and a challenge – strength in inception and drive and challenging as funding uncertainty and staff changes led to de-prioritisation of FRSIS by multi-agency partners. While there were multiple early champions of the program across multiple agencies and multiple layers of leadership, this waned substantially as the funding window came to a close, despite operational success.
- Sustainability – this evaluation indicated clear operational success, however the time-limited program window, due to short-term funding, did not allow for implementation maturity across all schools and at the governance level. Sustained commitment from the multiagency partners was lacking (at multiple levels of leadership).
- Embedding evaluation at the start would have enabled data-informed program maturation and provided the evidence base early enough to justify a business case for ongoing funding. Moreover, this was particularly true given the desire to satisfy the differing goals of each stakeholder and the delays experienced in multi-agency co-design and delays in ethical approval. Even with multiple data sources and multiple ethical approvals, some data could not have been effectively drawn or linked as this capacity had not been baked into the program. This would have been an overinvestment for the pilot but highlights government data linkage and analytic limitations at the time.
- Collaborative and multi-agency approaches to addressing the integration of health and social care in a person-centred fashion hold great promise and policies often state this intent. However, these approaches surface inconsistent and sometimes conflicting priorities and objectives, which should be recognised and addressed at operational and strategic levels.

## Limitations

This evaluation was hampered by three main issues. Firstly, interagency data linkage was not supported by recording procedures, eliminating the systematic capture of full client journeys and related outcomes. Secondly, the participant response rate was poor. The evaluation experienced delays in workshop scheduling and ethical approval (four separate approvals across health, education, and university, and a site-specific approval system-related delay). This led to data collection coincident with a busy time for schools and was closer to the funding deadline. Had more participants been recruited, a broader range of views may have been expressed. Whilst voluntary participation may have selected those with a positive view, the anonymity of the survey, provided an avenue for participants to express negative views safely. Thirdly, the experience of the service users was not directly gathered, following the advice from the stakeholders and consideration of the families’ welfare.

## Conclusion

FRSIS appeared to be a program based on a promising concept that was well supported by frontline staff. There was good uptake of the program addressing the engagement gap experienced by the community version of FRS, enabling early intervention for families with complex and unmet needs to receive broad-ranging support, with the hope of avoiding formal dealings with child protection agencies. The commitment of these staff ensured that the program functioned well and delivered benefits to the families and schools served. The flexibility of the program to deal with any problem and being open to any family in need within the school contributed to its success.

The dominance of funding uncertainty and waning commitment from the oversight Working Group was disappointing given the operational success. The Central Coast FRSIS program showed the concept can be successfully adapted and implemented in multiple school jurisdictions, enabling consideration of wider uptake in NSW schools to effectively support children and families with complex and unmet needs in an integrated fashion.
